# Enhancing seizure prediction using a DC-SA-EBiLSTM framework with self-attention mechanism

**DOI:** 10.3389/fnins.2026.1856135

**Published:** 2026-06-05

**Authors:** Shunyun Wang, Jincan Zhang, Wenna Chen, Fei Xiang, Hongwei Jiang, Ganqin Du

**Affiliations:** 1 College of Information Engineering, Henan University of Science and Technology, Luoyang, China; 2 The First Affiliated Hospital, and College of Clinical Medicine of Henan University of Science and Technology, Luoyang, China

**Keywords:** electroencephalography, feature extraction, hybrid model, seizure prediction, self-attention

## Abstract

**Background:**

Accurately predicting seizures remains challenging. With advances in smart medical technology, EEG-based monitoring has become essential. This study aims to improve prediction accuracy using a hybrid framework that models multiscale EEG characteristics.

**Methods:**

EEG signals are decomposed into multiple sub-bands using the Discrete Wavelet Transform, and representative time-frequency and nonlinear features are extracted. These features are fed into a channel-centric model integrating depthwise separable convolution, self-attention, and an enhanced bidirectional long short-term memory network (DC-SA-EBiLSTM). The architecture integrates depthwise separable convolution for local spatial feature extraction, multi-head self-attention for global inter-channel dependencies, and an enhanced BiLSTM for channel-wise sequence modeling. The proposed method was evaluated on the CHB-MIT dataset using a 10-fold cross-validation protocol. An event-level leave-one-seizure-event-out validation was also conducted to assess alarm-based prediction performance.

**Results:**

The proposed approach achieved an average accuracy of 95.89%, sensitivity of 96.70%, specificity of 95.48%, and AUC of 99.02%. In the event-level validation, the model achieved an event sensitivity of 95.96%, an average false alarm rate of 0.316 FPR/h, and a mean early warning time of 30.52 min.

**Conclusion:**

The DC-SA-EBiLSTM framework effectively captures local and global inter-channel dependencies and provides a feature-driven approach for patient-specific preictal state prediction, showing potential for EEG-based seizure prediction.

## 1 Introduction

Epilepsy is a chronic neurological disease characterized by recurrent seizures that significantly impact quality of life (Ouyang et al., 2020). Patients often experience sudden, uncontrollable convulsions, and in severe cases, loss of consciousness. The abrupt and unpredictable nature of seizures not only causes emotional anxiety and helplessness, but can also be life-threatening at critical moments, especially when driving or performing other activities that require a high degree of concentration. Studies have shown that individuals with epilepsy are at an elevated risk of sudden unexpected death in epilepsy (Jha et al., 2021), which is a leading cause of mortality among epilepsy patients (Liu et al., 2020; Varnosfaderani et al., 2021). Research indicated that a reliable method for predicting seizures could help patients plan daily activities in advance, thereby improving their mental health and overall quality of life (Leguia et al., 2023). Therefore, accurate seizure prediction holds immense significance in mitigating risks, enhancing treatment outcomes, and improving the wellbeing of individuals with epilepsy.

Electroencephalography (EEG) is an effective and intuitive tool widely used to record brain activity during various physiological states (Siddiqui and Islam, 2016). EEG signals are generally divided into four phases: preictal, ictal, postictal, and interictal. Among these, the preictal phase is considered crucial for seizure prediction. Many studies suggest a transition period lasting from minutes to hours before a seizure (Abdelhameed and Bayoumi, 2018), which is pivotal for early seizure prediction. As Proix et al. (2021) observed, seizures are not entirely random, and periodic brain activity patterns can be utilized for preemptive prediction. Seizure prediction research focuses on identifying the preictal state through EEG signals, framing the task as a binary classification problem that distinguishes between preictal and interictal phases. When the EEG signal is classified as preictal, it signifies an impending seizure, enabling early warnings and proactive measures to mitigate potential harm (Yang et al., 2021).

Over the past few decades, significant progress has been made in seizure prediction research based on EEG signals. These studies primarily focus on two aspects: the extraction of representative features from the complex and diverse EEG signals, and the subsequent analysis and classification of these features. The original EEG signal is a kind of time series data, which directly depict the waveform changes of brain waves in the time domain. Traditional time-domain features, such as Standard Deviation (STD), kurtosis, mean and variance, can reflect temporal changes in the signal (Kaur et al., 2021; Pawar and Chougule, 2021; Hussein et al., 2022; Shoeibi et al., 2022). Siddiqui et al. (2019) extracted the maximum, mean, kurtosis, and skewness as features to detect seizures. However, time-domain features alone are insufficient to capture the frequency domain information embedded in EEG signals (Sone and Beheshti, 2021). It is a common method to transform the EEG signal from the time domain to the frequency domain through the Fourier Transform (FT), enabling the division of signals into distinct frequency bands. Widely used frequency domain features such as Power Spectral Density (PSD), band energy, spectral centroid, spectral moment, and spectral skewness (Aslam et al., 2022; Zambrana-Vinaroz et al., 2022). Recent advancements in seizure prediction research have aimed to capture the simultaneous temporal and spectral dynamics of EEG signals. Techniques such as Short-Time Fourier Transform (STFT), Empirical Mode Decomposition (EMD) (Humairani et al., 2021), Hilbert transform (Büyükçakır et al., 2020) and Wavelet Transform (WT) (Höller et al., 2020) have been employed to more precisely characterize EEG signal dynamics. For example, Cheng et al. (2021) used a DB4 WT to extract wavelet energy, revealing the time-frequency characteristics of EEG signals. Additionally, nonlinear features like approximate entropy, Fractal Dimension (FD) and Lyapunov exponent have been used to analyze the complexity and nonlinearity of EEG signals, significantly enhancing seizure prediction performance. Recently, fractal dimensionality has become increasingly popular in the study of seizures. Some scholars have studied the applicability of FD in predicting seizures (Brari and Belghith, 2021). Humairani et al. (2021) used FD in their research to measure the complexity of EEG signals due to their self-similarity and irregularity. Zhang Y. et al. (2020) used FD to analyze the Freiburg EEG dataset and finally achieved an average sensitivity of 90.42%. Furthermore, In the study of Zhang Y. et al. (2020), a rare nonlinear feature was proposed to reveal the directional flow of brain activity associated with epilepsy with nonlinear partial directional coherence. The study divided EEG signals into five sub-bands (Delta, Theta, Alpha, Beta, and Gamma) and achieved the highest accuracy of 89.2 ± 11.5% in the Beta sub-band.

After the characterization of EEG signals, designing an efficient classifier model is crucial for seizure prediction. A variety of classification models have been proposed for both binary and multivariate classification tasks. Mathematical statistical methods, such as Generalized Linear Models (Leguia et al., 2023), are particularly suitable for handling non-normally distributed data, while Kalman filtering can smooth and predict EEG signals in the presence of noise. Slimen et al. (2020) achieved seizure prediction by detecting the number of spikes in the EEG signal using a local maximum algorithm, with the maximum number of spikes in the interictal period serving as a threshold. In recent years, machine learning techniques have gained significant attention. Commonly used machine learning methods include Support Vector Machines (SVM), K-Nearest Neighbors (KNN), and Random Forests (RF) (Siddiqui et al., 2017). Usman et al. (2019) first applied a common-space mode filter to convert multi-channel EEG signals into a single-channel signal, then used WT for denoising, followed by Principal Component Analysis (PCA) to extract features, and finally classified the data using SVM. Siddiqui et al. (2020) integrated cost-sensitive learning with a RF classifier to mitigate the inherent imbalance in EEG data, employing recording duration as a weighted penalty to achieve high recall rates. Data imbalance is also a common challenge in biomedical signal modeling. For example, Yang et al. (2024a) proposed CECG-GAN to address ECG data imbalance by generating samples that approximate the original ECG distribution and by incorporating a Transformer architecture to improve generation efficiency.

Deep learning models, such as CNN and Long Short-Term Memory networks (LSTM), have proven to be highly effective in seizure prediction due to their capacity to process complex data. CNNs are capable of learning higher-order features from complex data, improving classification performance in seizure prediction and detection. Truong et al. (2017) designed a prediction method based on CNN, which first converted the original EEG signal into a two-dimensional matrix through a STFT, and fed the two-dimensional matrix as input to the CNN, and finally achieved an FPR of 0.17 and a sensitivity of 89.8%. In Fatma et al. (2023), the authors extracted multiclass features from EEG signals using an optimized CNN for classification and determined the optimal CNN weights based the Weighted Factor of the Shark Smell Optimization. Hussein et al. (2021) introduced a novel CNN architecture with a semi-dilated convolutional receptive field, supporting exponential growth in one dimension. Hu et al. (2023) applied a fifth-order Butterworth bandpass filter to preprocess EEG signals, followed by Discrete Wavelet Transform (DWT) for signal decomposition, and then employed transfer learning based on a hybrid transformer model for seizure prediction. LSTMs, known for their ability to handle long time-series data, have also been widely used in seizure prediction. Varnosfaderani et al. (2021) designed a two-layer LSTM model using the Sish activation function based on manually extracted time-frequency domain features, achieving sensitivity and accuracy of 86.8 and 85.1%, respectively, on the Melbourne dataset. Abdelhameed and Bayoumi (2018) proposed a semi-supervised deep learning system using a BiLSTM to capture the time dependencies within EEG signals, extending this to a two-dimensional deep convolutional autoencoder for classification. In Xu et al. (2022), the authors proposed convolutional Generative Adversarial Network (GAN) as the core generator based on Wasserstein distance, which improved the accuracy of epileptic seizure prediction. Recently, Attention Mechanisms (AMs) have gradually become a key technology in sequence modeling, which can effectively capture both long-term dependencies and local information within EEG signals (Vaswani et al., 2017). AMs process signals holistically, without being limited by the sequence length. Yan et al. (2022) employed a transformer network combined with AMs to address the model length limitation, achieving better results. Gao et al. (2022) utilized a multi-scale CNN with dilated convolution, coupled with a feature-weighted fusion strategy of AMs, achieving an average sensitivity of 93.3% on the CHB-MIT dataset.

Recent studies have also investigated hybrid and feature-fusion architectures for EEG-based seizure analysis. Yang et al. (2024b) proposed the LTY-CNN model, which integrates parallel multiscale convolution and multihead attention to capture EEG features at different scales. Ravi and Radhakrishnan (2024) developed a hybrid 1D CNN-BiLSTM model for epileptic seizure detection using multichannel EEG feature fusion, combining convolutional feature extraction with bidirectional recurrent modeling. Chen et al. (2023) proposed a CNN classifier based on feature fusion, in which DWT-derived entropy and statistical descriptors were selected using random forest and then classified by CNN. These studies indicate that combining complementary EEG features with hybrid deep learning modules is effective for seizure-related EEG analysis.

Conformer-style architectures provide another representative direction for combining convolutional operations with self-attention to capture local and global dependencies. These models are typically designed to learn representations from raw sequential signals or high-dimensional learned features. In contrast, the present study focuses on compact handcrafted descriptors extracted from DWT sub-bands. In addition, instead of using a conventional feed-forward module after attention, the proposed model introduces an enhanced BiLSTM block with residual projection, gated fusion, and layer normalization for contextual channel-wise feature fusion. Therefore, the proposed model is designed as a feature-driven hybrid framework rather than a direct raw-signal Conformer architecture.

Graph-based and topology-aware EEG models provide an effective way to characterize spatial or functional relationships among electrodes by representing EEG channels as graph nodes and modeling their interactions through predefined or learned connectivity structures (Jia et al., 2022; Wei et al., 2024). These methods are particularly useful for explicitly incorporating electrode topology or functional connectivity into EEG representation learning. In the present study, we adopt a different representation strategy without constructing an explicit electrode graph or adjacency matrix. Instead, DWT-based handcrafted descriptors are organized according to a fixed CHB-MIT bipolar montage order, and channel-wise dependencies are modeled through depthwise separable convolution, self-attention, and enhanced BiLSTM-based fusion.

Early identification of the preictal state is essential for seizure warning systems and long-term patient management. Although previous studies have explored handcrafted features, convolutional architectures, recurrent models, attention-based networks, and hybrid feature-fusion models, effectively integrating multiband spectral information, inter-channel dependencies, and contextual channel-wise representations remains challenging. To address this issue, we propose a DC-SA-EBiLSTM framework for patient-specific seizure prediction.

The main contributions of this study are threefold. First, a multiband feature-fusion strategy is developed to characterize preictal EEG dynamics by integrating DWT sub-band information with complementary energy, statistical, fractal, and entropy descriptors. Second, a feature-driven DC-SA-EBiLSTM architecture is developed to sequentially model local, global, and contextual relationships from multichannel EEG feature representations. Third, an enhanced BiLSTM-based fusion block is incorporated with residual projection, gated fusion, and layer normalization, and its contribution is evaluated through component-level ablation and statistical analysis.

The remainder of this paper is organized as follows: Section 2 introduces the datasets used in this study and details the proposed methods. Section 3 presents the experimental results and accompanying discussions. Section 4 concludes the paper, while Section 5 outlines the limitations of the experiments and future research directions.

## 2 Dataset and methods

**FIGURE 1:**
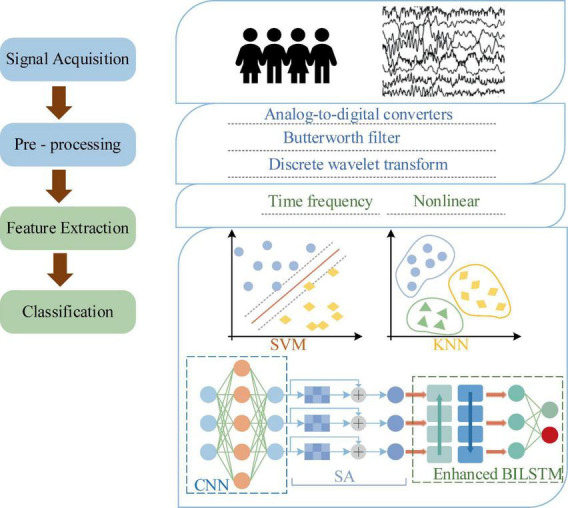
The overall structure of the proposed methodology.

This section outlines the steps involved in seizure prediction, and the framework of the entire process is shown in Figure 1. The preprocessing stage involves filtering the original multi-channel EEG signal and then decomposing it into sub-bands of different frequencies using DWT. After DWT processing, 6 sub-bands (A5, D1-D5) are generated, and four distinct features are extracted for each sub-band, including Higuchi FD, Fuzzy Entropy (FuEn), Root Mean Square (RMS) and STD. Consequently, each channel yields 24 features (6 sub-bands * 4 features per sub-band).

Since we used 18 EEG channels, the total number of features for each EEG segment is 432 (24 features* 18 channels). All features extracted from the sub-bands and channels are concatenated into a single feature vector. By organizing all these vectors, we get a structured two-dimensional feature dataset with a total of samples * 432 features, where each row represents the features of an EEG segment and each column corresponds to a specific extracted feature. In the classification phase, the extracted features are used to train and test our proposed DC-SA-EBiLSTM model.

### 2.1 Dataset description

In this work, we utilized the EEG signals of epilepsy patients from the CHB-MIT dataset, which was jointly created by Boston Children’s Hospital and the Massachusetts Institute of Technology. The CHB-MIT dataset is one of the most commonly used public datasets for epilepsy prediction and detection (Büyükçakır et al., 2020). It includes data from 23 patients (Andrade et al., 2024). EEG signals were collected according to international standards using the 10–20 electrode placement system, with a sampling frequency of 256 Hz and a resolution of 16 bits. For each patient, the EEG signals were stored in separate files, with each file containing between 9 and 42 recordings, each lasting 1 h or more. Additionally, the dataset provides annotation files containing important metadata, such as the recording start and end times, as well as annotations for seizure events, including the start and end times of each seizure. Table 1 provides a detailed description of the patient-specific information.

**TABLE 1:** Details of CHB-MIT patients.

Patient-ID	Gender F (female) M (male)	Age (years)	Number of seizures	Duration of seizure (s)	Recording duration (hh:mm:ss)
Chb-01	F	11	7	442	40:33:08
Chb-02	M	11	3	172	35:15:59
Chb-03	F	14	7	402	38:00:06
Chb-04	M	22	4	160	156:03:54
Chb-05	F	7	5	558	39:00:10
Chb-06	F	1.5	10	153	66:44:06
Chb-07	F	14.5	3	325	67:03:08
Chb-08	M	3.5	5	919	20:00:23
Chb-09	F	10	4	276	67:52:18
Chb-10	M	3	7	447	50:01:24
Chb-11	F	12	3	806	34:47:37
Chb-12	F	2	40	1,475	23:41:40
Chb-13	F	3	12	535	33:00:00
Chb-14	F	9	8	169	26:00:00
Chb-15	M	16	20	1,992	40:00:36
Chb-16	F	7	10	84	19:00:00
Chb-17	F	12	3	293	21:00:24
Chb-18	F	18	6	317	35:38:05
Chb-19	F	19	3	236	29:55:46
Chb-20	F	6	8	294	27:36:06
Chb-21	F	13	4	199	32:49:49
Chb-22	F	9	3	204	31:00:11
Chb-23	F	6	7	424	26:33:30
Total	-	-	-	10,882	961:38:20

### 2.2 Pre-processing

To predict seizures, we focus on EEG signals during the preictal and interictal periods. Based on previous studies that have characterized distinctive patterns in EEG signals (Büyükçakır et al., 2020), we define the preictal period as the interval from 33 to 3 min prior to seizure onset. The interictal period is defined as the interval between 4 h after a seizure and 4 h before the subsequent seizure, in order to minimize the potential influence of seizure-related activity on the signals. Both preictal and interictal data segments are further partitioned into non-overlapping 1 s windows.

During EEG acquisition, signals are often disturbed by factors such as electrode artifacts, eye movements, low-frequency drift, and power-line interference. To reduce slow baseline drift while preserving the spectral content required for subsequent multiresolution analysis, a fourth-order 0.5 Hz high-pass Butterworth filter was first applied to the raw EEG signals (Singh and Malhotra, 2022). Since the EEG recordings may also be contaminated by power-line interference, a narrow 60 Hz notch filter was further employed to suppress this component (Andrade et al., 2024). The preprocessed signals were then used for DWT-based sub-band decomposition and feature extraction.

**FIGURE 2:**
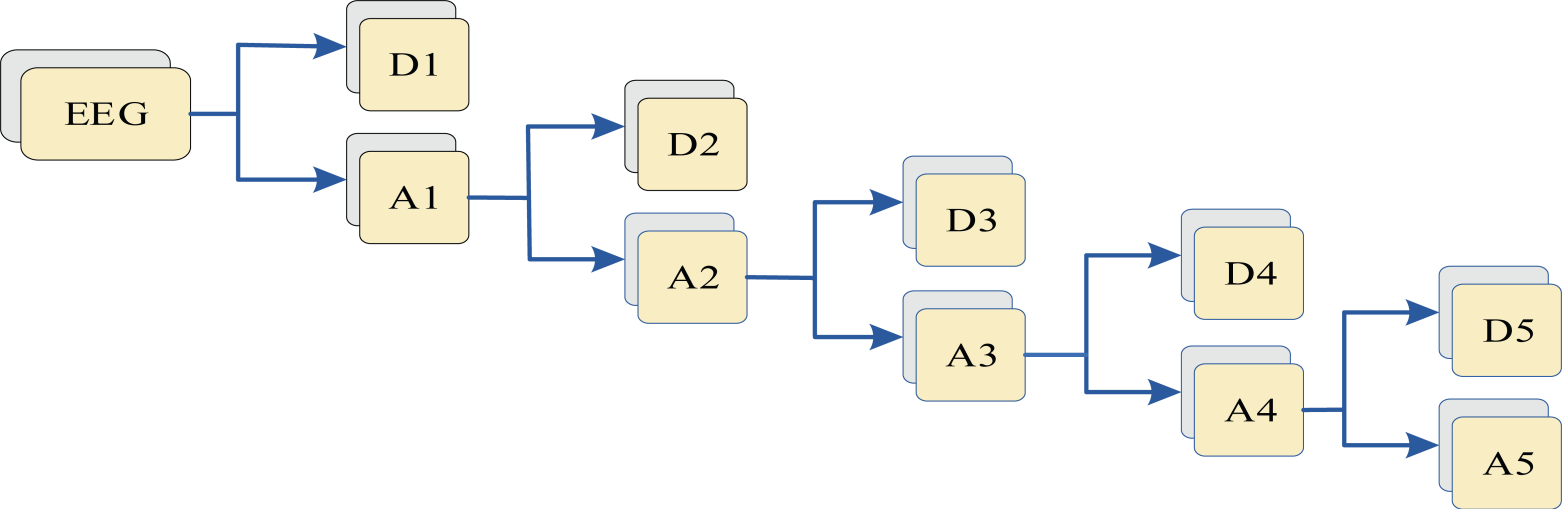
DWT decomposition flowchart.

WT has a wide range of applications in signal processing, and numerous studies have demonstrated its significant advantages in handling time-series data (Tokatly Latzer et al., 2020; Rasheed et al., 2021b; Zhang et al., 2024). Given that EEG signals are inherently non-stationary dynamic functions in the time domain, we used the Daubechies 4 (DB4) wavelet as the basis function and applied a five-level DWT to obtain multi-resolution sub-band signals. The DWT provides both time and frequency information, making it particularly suitable for processing EEG signals. A schematic representation of the wavelet decomposition process is shown in Figure 2. Through five-level DWT, the EEG signal was decomposed into six sub-bands: one fifth-level approximation coefficient (A5) and five detail coefficients (D1 to D5). With the EEG signals sampled at 256 Hz, the Nyquist frequency is 128 Hz. The nominal frequency ranges of the sub-bands are A5 (0–4 Hz), D5 (4–8 Hz), D4 (8–16 Hz), D3 (16–32 Hz), D2 (32–64 Hz), and D1 (64–128 Hz).

### 2.3 Feature extraction

The preprocessed EEG signals contain a vast amount of data with high dimensionality, making them unsuitable for direct input into classifiers. Therefore, it is essential to construct a feature set that is both low-dimensional and non-redundant (Aslam et al., 2022). The features described in this section are calculated on the individual sub-bands obtained after the EEG signal being decomposed via DWT. These features are then used to train a classification model to differentiate between preictal and interictal EEG signals. Compared to directly processing raw EEG signals, utilizing extracted features significantly reduces computational complexity and hardware requirements, making the system more efficient and cost-effective (Tsiouris et al., 2018; Varnosfaderani et al., 2021).

#### 2.3.1 Higuchi fractal dimension

The FD quantifies the complexity and self-similarity of a signal, making it be an essential feature for analyzing EEG signals. HFD, introduced by Higuchi in 1988, has been extensively used to study nonlinear time series such as EEG signals (Esteller et al., 2001). HFD focuses on constructing a virtual path and evaluating the fractal properties of the data by analyzing changes in path length. The steps of Higuchi’s fractal dimension algorithm are as follows:

Given a time series *x*(1), *x*(2),, *x*(*N*), the formula for calculating the average length *L*_*m*_(*k*) is generated as Equation 1 (Esteller et al., 2001).


$$L_{m}\,\left(k\right)=\frac{\left(N-1\right)}{\left\lfloor \frac{N-m}{k}\right\rfloor \,k}\,\sum_{i=1}^{\left\lfloor \left(N-m\right)/k\right\rfloor }\left|x\,\left(m+i\,k\right)-x\,\left(m+\left(i-1\right)\,k\right)\right|$$ (1)

where $\frac{\left(N-1\right)}{\left\lfloor \frac{N-m}{k}\right\rfloor \,k}$ is a normalization factor, *N* is the total length of the time series *x*,and *k* represents the delay factor. The average length across all *m* values from 1 to *k* is then computed as Equation 2 (Esteller et al., 2001).


$$L\,\left(k\right)=\sum_{m=1}^{k}L_{m}\,\left(k\right)$$ (2)

In this study, the maximum delay parameter kmax was set to half of the window length for each EEG segment. Since each segment contained 256 samples, kmax was set to 128.

#### 2.3.2 Fuzzy entropy

In biomedical signal processing, entropy is commonly used to quantify the degree of chaos and complexity within a signal. Entropy-based methods are particularly effective at indicating signal irregularities and are more robust against noise than many alternative techniques (Malekzadeh et al., 2021). FuEn extends the concept of traditional entropy, which measures signal complexity by considering the ambiguity of similar patterns within the signal.

For a time series signal *x*(*i*), the function Φ*^m^*(*n*,*r*) is then defined as Equation 3 (Acharya et al., 2019):


$$\Phi^{m}\,\left(n,r\right)=\frac{1}{N-m}\,\sum_{i=1}^{N-m}\left(\frac{1}{N-m-1}\,\sum_{j=1,j\neq i}^{N-m}D_{i\,j}^{m}\right)$$ (3)

FuEn is calculated as Equation 4.


$$F\,u\,E\,n\,\left(m,n,r,N\right)=-\ln\frac{\Phi^{m+1}\,\left(r\right)}{\Phi^{m}\,\left(r\right)}$$ (4)

For FuEn calculation, the embedding dimension m, fuzzy power n, and similarity tolerance r were set to 2, 1, and 0.2 × SD, respectively.

#### 2.3.3 Root mean square

Seizures are typically associated with significant abnormal fluctuations in electrical activity within the cerebral cortex. RMS has been shown to be an effective feature for quantifying energy changes in this electrophysiological activity. By calculating the RMS of an EEG signal, it becomes possible to quantify the overall energy level of the brain’s electrical activity over a specific time period. This provides critical insights into the neurophysiological state of the brain.

#### 2.3.4 Standard deviation

STD is a statistical feature that quantifies the degree of deviation between individual data points and the mean of a dataset, reflecting the dispersion or spread of the data. It is relatively robust against noise, making it be a reliable feature for statistical analysis. When combined with RMS and incorporated into the feature vector used in the classification model, it improves the accuracy of seizure prediction.

### 2.4 Proposed DC-SA-EBiLSTM model

**FIGURE 3:**
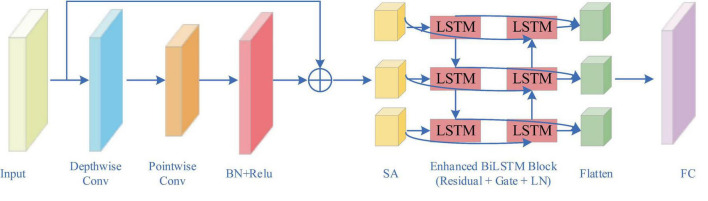
The proposed DC-SA-EBiLSTM architecture.

To effectively capture local correlations, global dependencies, and bidirectional contextual relationships among multi-channel EEG features, we propose a hybrid seizure prediction model termed DC-SA-EBiLSTM. Here, DC denotes depthwise separable convolution, SA refers to the self-attention mechanism, and EBiLSTM represents the enhanced bidirectional long short-term memory network. The proposed model uses the handcrafted features generated in the feature extraction stage as input. For each EEG segment, the extracted descriptors are stored as a 432-dimensional feature vector and then reorganized into a channel-wise feature matrix before model training. Based on this structured representation, the model performs hierarchical representation learning through channel embedding, local feature refinement, global dependency modeling, and contextual feature fusion. The overall architecture of the proposed model is illustrated in Figure 3.

#### 2.4.1 Channel embedding and depthwise separable convolution module

After feature extraction, each EEG segment is represented by a 432-dimensional feature vector. Since these handcrafted descriptors are derived from 18 selected EEG channels and each channel contributes 24 features, the feature vector is reorganized into a channel-wise feature matrix before being fed into the network. This structured representation preserves the channel-level organization of the extracted EEG descriptors and provides a suitable basis for subsequent feature fusion. The channel arrangement follows a predefined CHB-MIT bipolar montage order to ensure consistent input organization across patients. Specifically, the 18 selected bipolar channels were FP1-F7, F7-T7, T7-P7, P7-O1, FP1-F3, F3-C3, C3-P3, P3-O1, FP2-F4, F4-C4, C4-P4, P4-O2, FP2-F8, F8-T8, T8-P8, P8-O2, FZ-CZ, and CZ-PZ.

To improve the representational capacity of the handcrafted features, a channel embedding layer is first introduced to project the original descriptor of each channel into a higher-dimensional latent space. In this way, low-dimensional channel features can be transformed into more expressive representations before subsequent feature learning. Let *X* ∈ ℝ*^CF^*denote the reorganized feature matrix, where C is the number of channels and F is the number of features per channel. The channel embedding process is formulated in Equation 5:


$$H_{0}=X\,W_{e}+b_{e}$$ (5)

Where *W*_*e*_ ∈ ℝ*^Fd^*and *b*_*e*_ ∈ ℝ^*d*^ are learnable parameters, and *H*_0_ ∈ ℝ*^Cd^* denotes the embedded channel representation. In this study, the embedding dimension d is set to 64.

Based on the embedded representation in Equation 5, a depthwise separable convolution module is further employed to capture local inter-channel patterns. Compared with standard convolution, depthwise separable convolution decomposes the convolution process into a depthwise convolution and a pointwise convolution, which reduces computational complexity while preserving effective local feature extraction capability. Specifically, the depthwise convolution is used to extract local patterns from each embedding channel, whereas the pointwise convolution is adopted to integrate the resulting information across channels. The local feature extraction process can be expressed as Equation 6:


$$H_{d\,c}=P\,W\,C\,o\,n\,v\,\left(D\,W\,C\,o\,n\,v\,\left(H_{0}\right)\right)$$ (6)

Where *DWConv*(⋅)and *PWConv*(⋅) denote the depthwise and pointwise convolution operations, respectively.

To preserve the original embedded information while incorporating local structural patterns extracted by Equation 6, a residual connection is introduced between the convolutional output and the embedded feature representation. Accordingly, the output of the local feature extraction stage is defined in Equation 7:


$$H_{1}=H_{0}+D\,r\,o\,p\,o\,u\,t\,\left(H_{d\,c}\right)$$ (7)

Where *H_1_* denotes the refined feature representation after local inter-channel modeling. As indicated by Equations 5–7, the proposed module first enhances the original handcrafted descriptors through channel embedding and then captures local dependencies among neighboring EEG channels via depthwise separable convolution. This design allows the network to learn more informative channel-wise representations while maintaining stable information propagation for subsequent global dependency modeling.

#### 2.4.2 Self-attention module

Although the depthwise separable convolution module can effectively capture local inter-channel patterns, its receptive field remains limited for modeling long-range dependencies among EEG channels. To address this issue, a self-attention module is introduced after local feature extraction. Based on the refined representation obtained from the previous stage, a learnable positional encoding is incorporated to provide an auxiliary index reference for the predefined channel arrangement. The resulting features are then fed into a multi-head self-attention mechanism, which enables each channel-wise representation to interact with the others and provides a global view of relationships among the extracted EEG feature representations.

The core operation of the self-attention mechanism is defined in Equation 8:


$$A\,t\,t\,e\,n\,t\,i\,o\,n\,\left(Q,K,V\right)=S\,o\,f\,t\,m\,a\,x\,\left(\frac{Q\,K^{T}}{\sqrt{d_{k}}}\right)$$ (8)

Where *Q*, *K*, and *V* denote the query, key, and value matrices, respectively, and d_*k*_ is the scaling factor associated with the key dimension. As shown in Equation 8, the self-attention mechanism adaptively assigns larger weights to more informative channel interactions while suppressing less relevant dependencies.

After attention calculation, residual connections and a feed-forward network are further employed to refine the global feature representation and improve optimization stability. In this way, the self-attention module enables the proposed model to move beyond local convolutional perception and to capture long-range global dependencies across EEG channels, thereby providing more informative feature representations for the subsequent enhanced BiLSTM module.

#### 2.4.3 Enhanced BiLSTM module

To further enhance contextual fusion among channel-wise EEG feature representations, an enhanced bidirectional long short-term memory (EBiLSTM) module is introduced after the self-attention stage. In the proposed framework, the recurrent structure is applied to the predefined channel-wise feature matrix rather than to the raw temporal sampling axis. In this way, the model integrates contextual information across the fixed montage-based channel representation from both forward and backward directions.

Unlike a conventional BiLSTM block, the proposed EBiLSTM incorporates residual projection, gated fusion, and layer normalization to improve both representational capacity and training stability. Specifically, after bidirectional recurrent encoding, the input features are projected to the same dimensionality as the BiLSTM output, so that the original channel representation can be preserved through a residual branch. The gating vector is then generated by jointly considering the BiLSTM output and the projected input representation, as defined in Equation 9. Based on the resulting gating vector, the recurrent output and the residual branch are adaptively fused and subsequently normalized, as shown in Equation 10. Through this design, the proposed module can preserve discriminative input information while still benefiting from bidirectional dependency modeling.


$$G=\sigma \left(W_{g}\left[H_{B\,i\,L\,S\,T\,M};;H_{p\,r\,o\,j}\right]+b_{g}\right)$$ (9)

$$H_{4}=L\,N\,\left(G\,⨀H_{B\,i\,L\,S\,T\,M}+\left(1-G\right)\,⨀H_{p\,r\,o\,j}\right)$$ (10)

**FIGURE 4:**
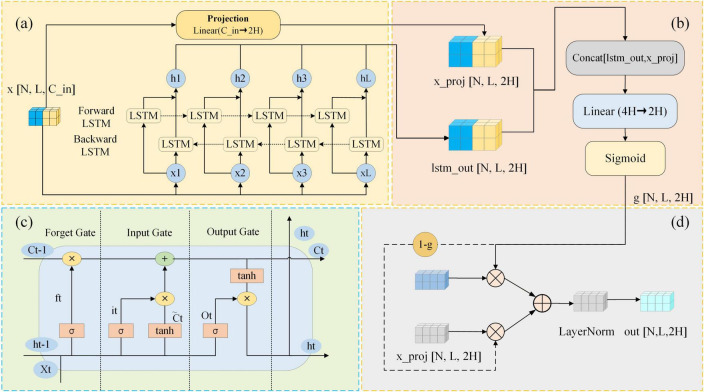
Detailed architecture of the proposed EBiLSTM block: **(a)** bidirectional recurrent encoding with a parallel projection branch; **(b)** joint gate generation from the concatenated recurrent output and projected input; **(c)** internal structure of a standard LSTM cell; and **(d)** adaptive gated fusion followed by layer normalization.

Where [H_BiLSTM_; H_proj_] denotes the concatenation of H_BiLSTM_ and H_proj_ along the last feature dimension, σ(⋅) denotes the sigmoid activation function, H_BiLS*TM*_ is the bidirectional recurrent output, H_proj_ is the projected input representation, G is the gating vector, ⨀ denotes element-wise multiplication, and LN(⋅) denotes layer normalization. The detailed architecture of the proposed EBiLSTM block is illustrated in Figure 4. Specifically, Figure 4a shows the bidirectional recurrent encoding together with the parallel projection branch, Figure 4b presents the generation of the gating vector from the concatenated recurrent output and projected input, Figure 4c depicts the internal structure of a standard LSTM cell for completeness, and Figure 4d illustrates the adaptive gated fusion followed by layer normalization.

Compared with a standard BiLSTM, the proposed EBiLSTM provides a more flexible and informative feature fusion mechanism. As shown in Figure 4, the residual projection branch helps retain the original feature semantics, the joint gating strategy in Equation 9 enables adaptive integration of recurrent and residual representations, and the fusion formulation in Equation 10 stabilizes feature propagation through layer normalization. Therefore, the enhanced design can generate more robust and discriminative channel-wise representations for the final classification task.

#### 2.4.4 Classification head

After contextual refinement by the proposed EBiLSTM module, the resulting high-level channel representations are flattened and fed into a fully connected classification head for final decision making. The classifier is composed of a linear layer, batch normalization, a ReLU activation function, dropout regularization, and an output layer. This design further integrates the learned representations and reduces overfitting, thereby enabling the final discrimination between preictal and interictal EEG segments.

For clarity, the detailed layer configuration, output dimensions, and key architectural hyperparameters of the proposed DC-SA-EBiLSTM model are summarized in Table 2.

**TABLE 2:** Overall architectural configuration and key hyperparameters of the proposed DC-SA-EBiLSTM model.

Module	Components	Output size	Key hyperparameters
Input reshaping	Reshape input feature vector	18 × 24	Number of channels = 18; features per channel = 24
Channel embedding	Linear projection	18 × 64	Embedding dimension = 64
Local feature extraction	Depthwise separable convolution block	18 × 64	Kernel size = 3; groups = 64; dropout = 0.2
Global dependency modeling	Positional encoding + multi-head self-attention + feed-forward network	18 × 64	Embed dimension = 64; number of heads = 4; FFN expansion ratio = 2; dropout = 0.2
Enhanced contextual modeling	Bidirectional LSTM + projection branch + gated fusion + LayerNorm	18 × 64	Hidden size = 32; number of layers = 1; bidirectional
Classification head	Two-layer fully connected classifier	2	Hidden dimension = 128; dropout = 0.3

### 2.5 Implementation and evaluation protocol

To evaluate patient-specific seizure prediction performance, 10-fold cross-validation was conducted separately for each included patient. For each patient, the preictal and interictal windows were partitioned into 10-folds. In each iteration, one-fold was used for testing, one part of the remaining data was used for validation, and the remaining folds were used for model training. The final patient-level performance was obtained by averaging the results across the 10 test folds. Standardization was performed within each fold: the scaler was fitted only on the training set and then applied to the validation and test sets.

**FIGURE 5:**
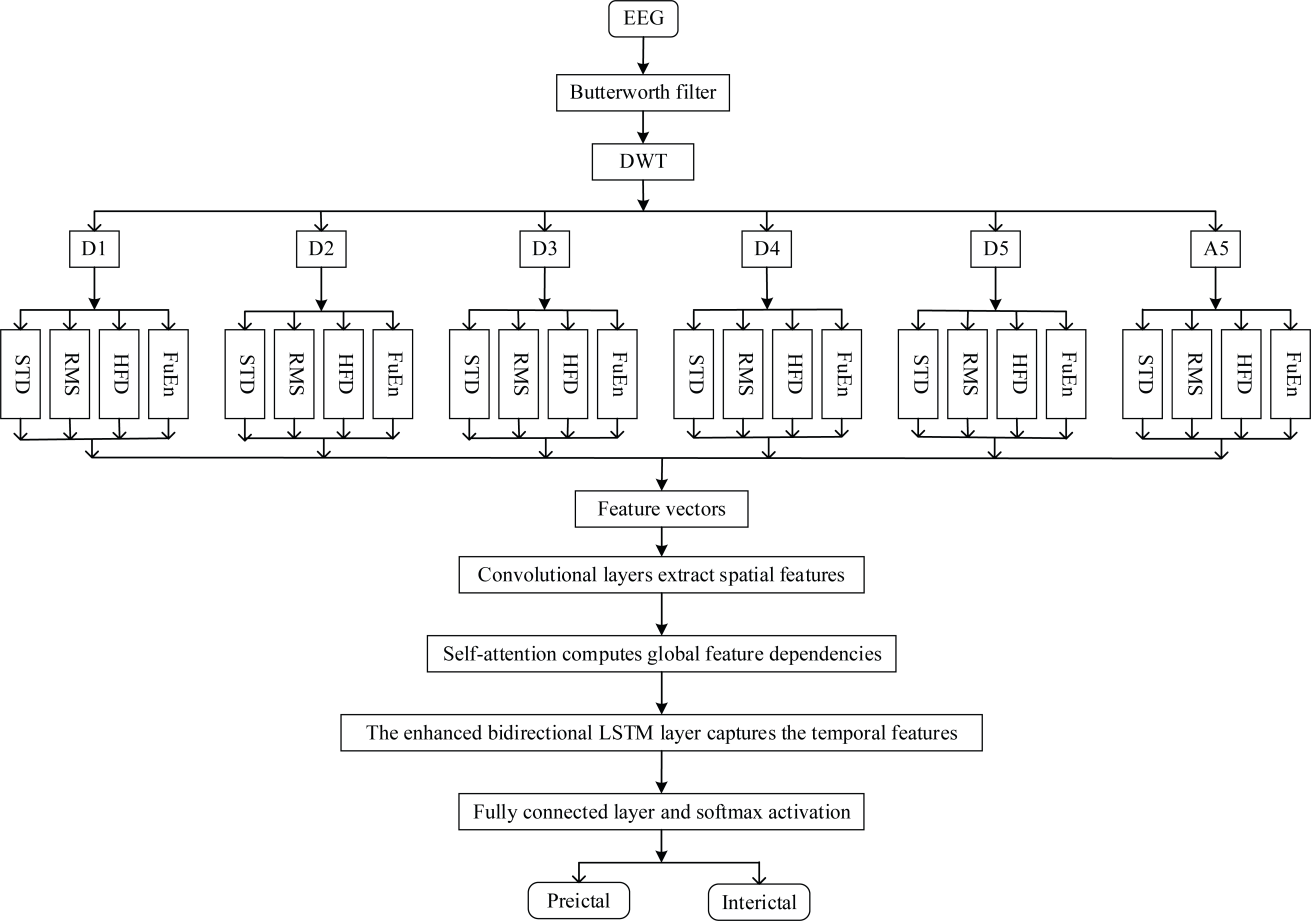
Flowchart of the proposed method.

To mitigate class imbalance in the patient-specific 10-fold experiments, the training set was processed using a 1:2 majority-class undersampling strategy. The validation and test sets were not undersampled and retained their original class distributions. This design was used to reduce the dominance of interictal samples during training while keeping model selection and final testing closer to the original data distribution. The flowchart of the proposed method in this study is shown in Figure 5.

All neural network models were trained using the Adam optimizer with a learning rate of 1 × 10^–3^, weight decay of 1 × 10^–4^, and a batch size of 128. The maximum number of training epochs was set to 100. Early stopping was applied with a patience of 30 epochs based on validation performance. A step learning-rate scheduler was used with a step size of 80 epochs and a decay factor of 0.1.

For fair comparison, all baseline models were trained and evaluated using the same preprocessing pipeline, feature set, patient-specific folds, standardization procedure, and evaluation metrics. The SVM used an RBF kernel with C = 10 and gamma = scale. The KNN classifier used k = 5 with distance weighting. The neural baselines, including CNN, LSTM, BiLSTM, SA-EBiLSTM, and CNN-EBiLSTM, were trained under the same optimization settings as the proposed model.

In addition to the patient-specific 10-fold evaluation, an event-level leave-one-seizure-event-out validation was performed to provide a stricter prediction-oriented assessment. This analysis was conducted on patients with at least three valid seizure events and sufficient clean interictal blocks. In each fold, one seizure event was held out for testing. One remaining seizure event was used for validation, and the other events were used for training. Class imbalance was handled using a class-weighted cross-entropy loss. The event-level evaluation reported per-seizure sensitivity, false positives per hour (FPR/h), and early warning time. The definitions of these metrics and the statistical procedures are described in section 3.1.

All experiments were conducted on a Windows 11 workstation equipped with an NVIDIA GeForce RTX 3080 GPU. The software environment was based on Python 3.12.6 and PyTorch 2.5.1 with CUDA 11.8.

## 3 Results and discussion

### 3.1 Performance measures and statistical analysis

To evaluate the performance of the proposed method, classification metrics and event-level seizure prediction metrics were used. In the patient-specific 10-fold cross-validation experiments, Accuracy (ACC), Sensitivity (SEN), Specificity (SPE), and Area Under the Receiver Operating Characteristic Curve (AUC) were reported. ACC reflects the overall proportion of correctly classified samples, SEN measures the ability to correctly identify preictal samples, and SPE evaluates the ability to correctly identify interictal samples. These metrics are defined in Equations 11–13.


$$A\,C\,C=\frac{T\,P+T\,N}{T\,P+T\,N+F\,P+F\,N}$$ (11)

$$S\,E\,N=\frac{T\,P}{T\,P+F\,N}$$ (12)

$$S\,P\,E=\frac{T\,N}{T\,N+F\,P}$$ (13)

where TP and TN denote the numbers of correctly classified preictal and interictal samples, respectively. FP denotes the number of interictal samples incorrectly classified as preictal, and FN denotes the number of preictal samples incorrectly classified as interictal. AUC was used to evaluate the discriminative ability of the model across different decision thresholds.

For the event-level leave-one-seizure-event-out evaluation, alarm generation was based on a short-term voting strategy. Specifically, a sliding alarm window containing 30 consecutive 1-s EEG segments was used, and an alarm was generated when at least 10 of the 30 segments were classified as preictal. This strategy was used to reduce isolated false-positive segment predictions before calculating event-level metrics. Per-seizure sensitivity was defined as the proportion of test seizure events for which at least one valid alarm was generated during the preictal interval. FPR/h was calculated as the number of false alarms divided by the total duration of test interictal recordings in hours. Early warning time was defined as the time interval between seizure onset and the first valid alarm within the preictal interval.

For statistical analysis, fold-level results were first averaged within each patient to obtain patient-level performance values. The overall results were then summarized across patients as mean ± standard deviation. The 95% bootstrap confidence intervals were estimated by resampling patients with replacement. For baseline comparison and component-level ablation, pairwise comparisons between the full DC-SA-EBiLSTM model and each competing model were performed using the Wilcoxon signed-rank test based on patient-level AUC values. Benjamini-Hochberg false discovery rate correction was applied to adjust for multiple comparisons, and the corrected *p*-values are denoted as p_adj in Tables 3, 4. Paired Cohen’s d was reported as the effect size relative to the full DC-SA-EBiLSTM model. In addition, Friedman tests followed by Nemenyi *post-hoc* ranking were used to assess overall rank differences among multiple models based on patient-level AUC values.

**TABLE 3:** Comparison with baseline models on the CHB-MIT dataset.

Model	ACC (%)	SEN (%)	SPE (%)	AUC (%)	95%CI	P_adj	Cohen’s *d*
SVM	92.03 ± 9.66	95.91 ± 2.46	90.36 ± 12.70	97.71 ± 3.27	[96.23, 98.94]	6.67 × 10^–6^	0.712
KNN	77.94 ± 15.04	91.73 ± 10.41	74.18 ± 20.04	90.88 ± 8.78	[87.02, 94.42]	6.67 × 10^–6^	1.085
CNN	90.93 ± 10.50	95.34 ± 2.59	89.04 ± 13.79	97.33 ± 3.60	[95.73, 98.70]	6.67 × 10^–6^	0.793
LSTM	94.92 ± 6.87	96.30 ± 2.63	94.26 ± 8.22	98.73 ± 2.01	[97.82, 99.46]	9.18 × 10^–5^	0.658
BiLSTM	95.10 ± 6.11	96.27 ± 2.94	94.41 ± 7.39	98.73 ± 2.03	[97.81, 99.46]	1.47 × 10^–4^	0.611
SA-EBiLSTM	95.83 ± 5.33	96.56 ± 3.03	95.37 ± 6.12	98.95 ± 1.85	[98.08, 99.60]	6.28 × 10^–3^	0.526
CNN-EBiLSTM	95.47 ± 6.33	96.71 ± 2.46	94.90 ± 7.46	98.93 ± 1.79	[98.11, 99.57]	2.82 × 10^–4^	0.553
**DC-SA-EBiLSTM**	95.89 ± 5.55	**96.70 ± 2.68**	**95.48 ± 6.36**	**99.02 ± 1.72**	**[98.22, 99.62]**	**–**	**–**

Bold values indicate the results of the method proposed in this paper.

**TABLE 4:** Component-level ablation study of the proposed model.

Model	ACC (%)	SEN (%)	SPE (%)	AUC (%)	95%CI	P_adj	Cohen’s *d*
**Full DC-SA-EBiLSTM**	**95.89 ± 5.55**	**96.70 ± 2.68**	**95.48 ± 6.36**	**99.02 ± 1.72**	**[98.22, 99.62]**	**–**	**–**
DC-SA-BiLSTM	95.53 ± 6.35	96.62 ± 2.73	95.03 ± 7.39	98.92 ± 1.83	[98.07, 99.57]	4.67 × 10^–05^	0.707
Without gated fusion	95.75 ± 5.73	96.71 ± 2.63	95.24 ± 6.68	98.97 ± 1.77	[98.15, 99.60]	2.8 × 10^–03^	0.602
Without residual projection branch	95.59 ± 5.54	96.55 ± 2.93	95.03 ± 6.51	98.89 ± 1.88	[98.02, 99.56]	1.10 × 10^–04^	0.660
Without LayerNorm	95.83 ± 5.50	96.58 ± 2.87	95.39 ± 6.32	98.96 ± 1.81	[98.12, 99.60]	6.47 × 10^–03^	0.559
Without self-attention	95.44 ± 6.48	96.58 ± 2.54	94.90 ± 7.61	98.91 ± 1.86	[98.05, 99.57]	1.22 × 10^–04^	0.699
DSConv replaced by standard Conv1d	95.78 ± 5.51	96.77 ± 2.69	95.28 ± 6.41	98.98 ± 1.75	[98.17, 99.60]	3.19 × 10^–02^	0.414
Without positional encoding	95.91 ± 5.07	96.36 ± 3.19	95.54 ± 5.75	98.95 ± 1.79	[98.12, 99.58]	5.25 × 10^–03^	0.655

Bold values indicate the results of the method proposed in this paper.

### 3.2 Results

**FIGURE 6:**
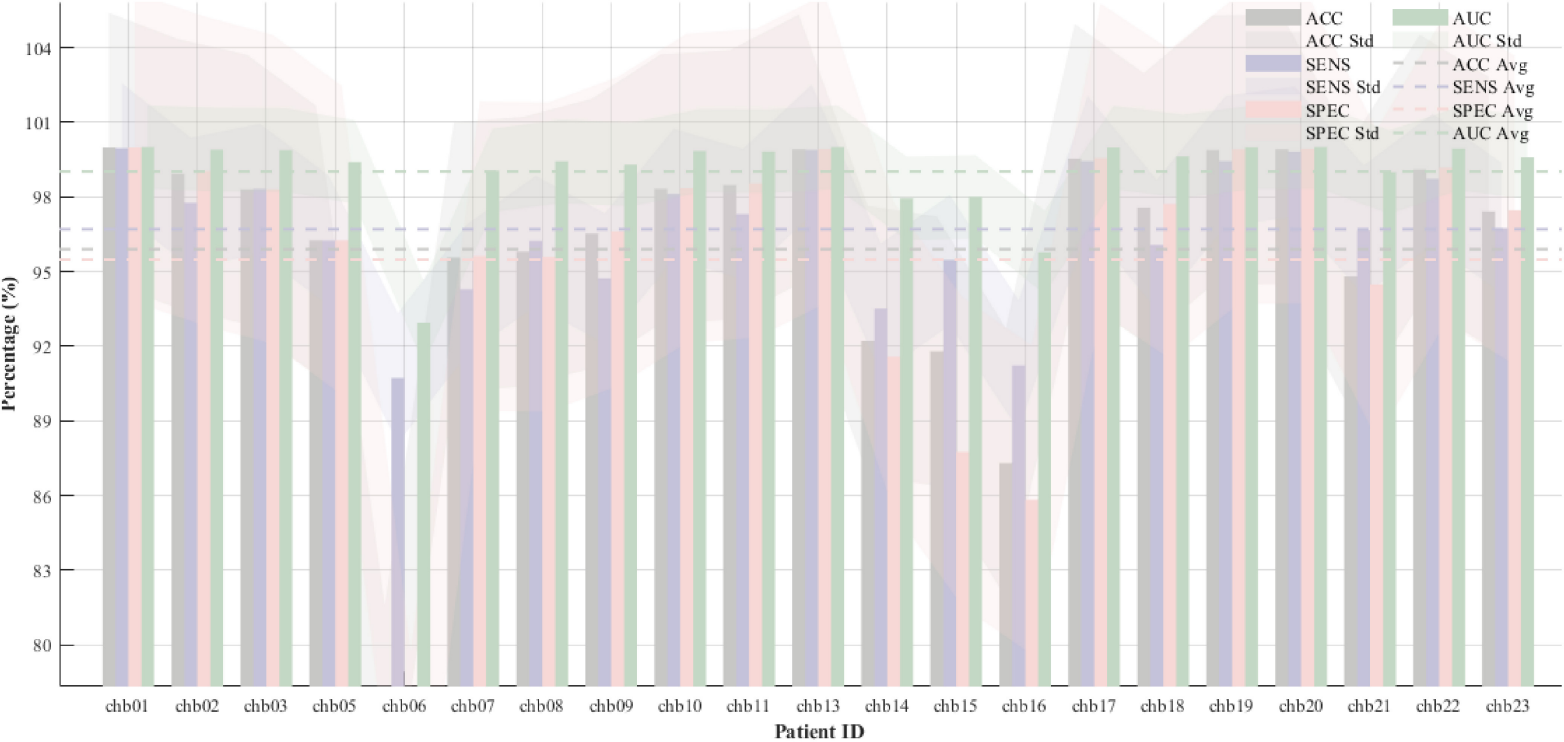
Patient-wise classification performance on the CHB-MIT dataset.

As shown in Table 5, the proposed DC-SA-EBiLSTM model achieved overall favorable patient-specific performance across the included CHB-MIT patients. Across 21 patients, the average ACC, SEN, SPE, and AUC were 95.89 ± 5.55%, 96.70 ± 2.68%, 95.48 ± 6.36%, and 99.02 ± 1.72%, respectively. The corresponding 95% bootstrap confidence intervals were 93.34–97.85% for ACC, 95.55–97.77% for SEN, 92.57–97.76% for SPE, and 98.22–99.62% for AUC. These results indicate that the proposed model maintained high sensitivity and discriminative ability across the included patients. The patient-wise distributions of ACC, SEN, SPE, and AUC are further illustrated in Figure 6.

**TABLE 5:** The classification metrics on the CHB-MIT dataset.

Patient-ID	ACC (%)	SEN (%)	SPE (%)	AUC (%)
Chb-01	99.98 ± 0.02	99.95 ± 0.07	99.98 ± 0.02	100.00 ± 0.00
Chb-02	98.93 ± 0.16	97.76 ± 0.67	99.05 ± 0.18	99.90 ± 0.03
Chb-03	98.30 ± 0.21	98.32 ± 0.59	98.30 ± 0.33	99.88 ± 0.02
Chb-05	96.25 ± 0.48	96.24 ± 1.05	96.25 ± 0.63	99.40 ± 0.10
Chb-06	76.18 ± 6.27	90.72 ± 5.56	73.53 ± 8.41	92.95 ± 0.30
Chb-07	95.57 ± 0.76	94.28 ± 1.44	95.62 ± 0.83	99.06 ± 0.15
Chb-08	95.80 ± 0.35	96.23 ± 0.90	95.59 ± 0.79	99.43 ± 0.09
Chb-09	96.53 ± 0.63	94.72 ± 0.84	96.61 ± 0.67	99.30 ± 0.13
Chb-10	98.32 ± 0.24	98.12 ± 0.53	98.35 ± 0.33	99.85 ± 0.03
Chb-11	98.48 ± 0.42	97.31 ± 1.54	98.54 ± 0.43	99.83 ± 0.09
Chb-13	99.91 ± 0.06	99.89 ± 0.09	99.92 ± 0.06	100.00 ± 0.00
Chb-14	92.22 ± 0.61	93.51 ± 1.36	91.58 ± 1.28	97.94 ± 0.16
Chb-15	91.80 ± 0.68	95.46 ± 1.63	87.75 ± 1.58	98.00 ± 0.42
Chb-16	87.31 ± 1.47	91.23 ± 1.76	85.83 ± 2.35	95.77 ± 0.54
Chb-17	99.53 ± 0.17	99.44 ± 0.26	99.55 ± 0.20	99.98 ± 0.01
Chb-18	97.56 ± 0.26	96.07 ± 0.87	97.72 ± 0.24	99.63 ± 0.09
Chb-19	99.88 ± 0.05	99.44 ± 0.56	99.91 ± 0.07	99.99 ± 0.03
Chb-20	99.91 ± 0.04	99.82 ± 0.12	99.93 ± 0.04	100.00 ± 0.01
Chb-21	94.81 ± 0.77	96.65 ± 0.92	94.48 ± 0.90	98.99 ± 0.19
Chb-22	99.10 ± 0.31	98.72 ± 0.49	99.17 ± 0.38	99.94 ± 0.04
Chb-23	97.41 ± 0.46	96.75 ± 1.19	97.46 ± 0.50	99.59 ± 0.12
Mean ± SD	95.89 ± 5.55	96.70 ± 2.68	95.48 ± 6.36	99.02 ± 1.72
95% Bootstrap CI	[93.34, 97.85]	[95.55, 97.77]	[92.57, 97.76]	[98.22, 99.62]

At the individual-patient level, most patients achieved high prediction performance. Chb-01, Chb-13, and Chb-20 showed AUC values close to 100%, with ACC, SEN, and SPE all above 99%. Several other patients, including Chb-02, Chb-03, Chb-10, Chb-17, Chb-19, and Chb-22, also achieved AUC values above 99.8%. These results suggest that the proposed model could effectively distinguish preictal and interictal states in a large proportion of patients.

Inter-patient variability was still observed, especially in Chb-06, Chb-14, Chb-15, Chb-16, and Chb-21. Chb-06 was the most challenging case, with the lowest ACC of 76.18% and SPE of 73.53%, although its SEN remained 90.72% and its AUC reached 92.95%. This pattern suggests that the model could still identify most preictal samples, but a relatively large proportion of interictal samples was misclassified as preictal. Chb-16 showed a similar trend, with an ACC of 87.31%, SEN of 91.23%, SPE of 85.83%, and AUC of 95.77%, indicating that the reduced performance was mainly associated with lower specificity rather than a complete loss of discriminative ability.

Chb-14, Chb-15, and Chb-21 also showed lower performance than the best-performing subjects, but their AUC values remained high. For Chb-14, the ACC, SEN, SPE, and AUC were 92.22, 93.51, 91.58, and 97.94%, respectively. For Chb-15, the corresponding values were 91.80, 95.46, 87.75, and 98.00%, respectively. Chb-21 achieved 94.81% ACC, 96.65% SEN, 94.48% SPE, and 98.99% AUC. The lower SPE compared with SEN in these relatively challenging subjects indicates that false alarms in interictal windows contributed more to the performance reduction than missed preictal detections.

**FIGURE 7:**
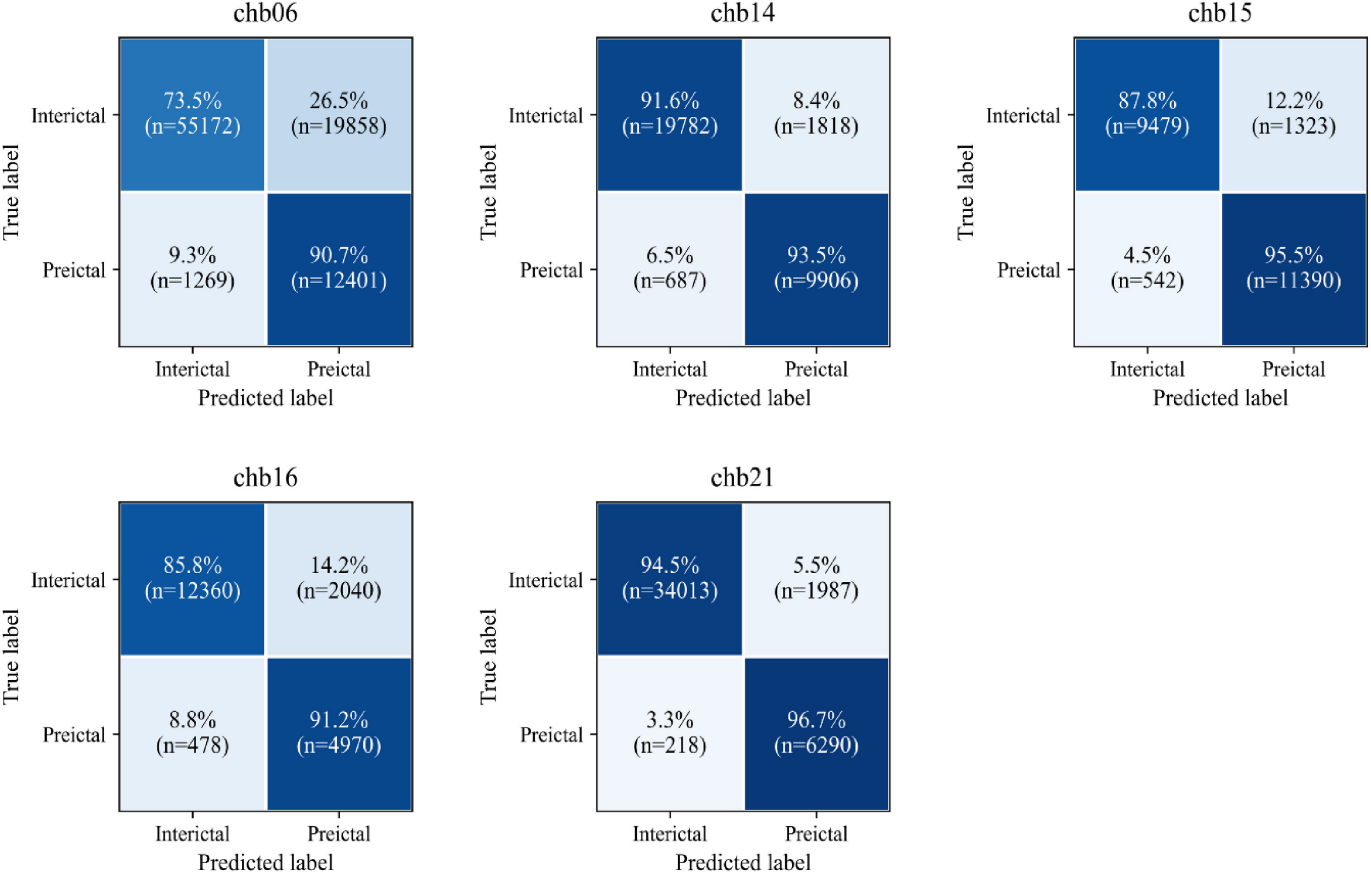
Confusion matrices of relatively challenging patients.

To further examine the error patterns in these relatively challenging subjects, confusion matrices were accumulated across the 10 test folds, as shown in Figure 7. Chb-06 had the highest FP rate, with 26.5% of interictal samples classified as preictal, while its FN rate was 9.3%. Chb-16 also showed an FP-dominant pattern, with an FP rate of 14.2% and an FN rate of 8.8%. For Chb-15 and Chb-21, the FP rates were 12.2 and 5.5%, respectively, whereas the corresponding FN rates were 4.5 and 3.3%. Chb-14 showed a more moderate but similar pattern, with an FP rate of 8.4% and an FN rate of 6.5%. These results indicate that the reduced ACC or SPE in these subjects was mainly associated with false-positive predictions in interictal windows rather than missed preictal detections.

As shown in Table 3, the proposed DC-SA-EBiLSTM model was compared with traditional machine learning classifiers and neural network baselines under the same preprocessing and evaluation protocol. The proposed model achieved the highest ACC, SPE, and AUC among all compared methods, while its SEN was comparable to the highest SEN obtained by CNN-EBiLSTM. Its AUC 95% CI was 98.22–99.62%, which was higher and relatively narrow compared with most baseline models, indicating stable discriminative performance across patients. Based on patient-level AUC values, the proposed model showed statistically significant differences from all baseline models after Benjamini-Hochberg FDR correction. The paired Cohen’s d values ranged from 0.526 to 1.085, corresponding to moderate-to-large effect sizes, with the largest effect observed against KNN. In addition, the Friedman test indicated a significant overall difference among the baseline models based on patient-level AUC values (χ^2^ = 120.698, *p* = 5.481 × 10^–23^), and the Nemenyi post-hoc ranking placed DC-SA-EBiLSTM first with the best average rank of 1.905.

Among the traditional classifiers, SVM achieved better overall performance than KNN, whereas KNN showed substantially lower ACC, SPE, and AUC, suggesting that simple distance-based classification was less suitable for the extracted high-dimensional EEG features. Compared with single-stream neural baselines such as CNN, LSTM, and BiLSTM, the hybrid variants incorporating attention or convolutional feature refinement achieved stronger overall performance. The full DC-SA-EBiLSTM further integrated depthwise separable convolution, self-attention, and enhanced BiLSTM-based contextual fusion, resulting in the best AUC and the most favorable overall statistical comparison in Table 3.

To further evaluate the contribution of each architectural component, a component-level ablation study was conducted, as shown in Table 4. Compared with the full DC-SA-EBiLSTM model, all ablated variants showed lower AUC values, and all AUC differences remained statistically significant after FDR correction. In addition to AUC, the full model also maintained the best or near-best balance among ACC, SEN, and SPE, indicating that the complete architecture provided balanced prediction performance across multiple metrics. The Friedman test also indicated a significant overall difference among the full model and its ablated variants based on patient-level AUC values (χ^2^ = 52.921, *p* = 3.843 × 10^–9^), and the Nemenyi *post-hoc* ranking placed the full DC-SA-EBiLSTM first with the best average rank of 2.619.

Among the ablated variants, removing the residual projection branch caused a clear reduction in overall classification performance. The ACC decreased from 95.89 to 95.59%, SPE decreased from 95.48 to 95.03%, and AUC decreased from 99.02 to 98.89%. This suggests that the residual projection branch helped preserve useful embedded channel-wise information during bidirectional contextual fusion. Removing self-attention also reduced ACC to 95.44%, SPE to 94.90%, and AUC to 98.91%, indicating that global interaction modeling among channel-wise feature representations contributed to both discriminative ability and interictal classification. Replacing EBiLSTM with a standard BiLSTM reduced ACC to 95.53% and AUC to 98.92%, supporting the effectiveness of the enhanced BiLSTM design over a conventional recurrent block.

The remaining components also contributed to the overall framework. Removing gated fusion yielded comparable SEN but reduced ACC, SPE, and AUC, suggesting that adaptive fusion between recurrent and residual representations was beneficial for maintaining overall classification balance. Removing LayerNorm led to slightly lower SEN and AUC, indicating that normalization helped stabilize representation learning. Replacing DSConv with standard Conv1d produced a similar SEN but lower ACC, SPE, and AUC, supporting the use of DSConv for effective local feature refinement. Removing positional encoding slightly increased ACC and SPE but reduced SEN and AUC, suggesting that the auxiliary channel-index embedding may help preserve sensitivity and discriminative ability in the attention module.

The standard deviations also provide information about inter-patient variability. Variants such as “Without self-attention” and “EBiLSTM replaced by standard BiLSTM” showed larger variability in ACC or SPE than the full model, suggesting less stable performance across patients. In contrast, the full DC-SA-EBiLSTM maintained a favorable balance between mean performance and inter-patient variability. The paired Cohen’s d values ranged from 0.414 to 0.707, indicating measurable component effects across the ablation variants. Overall, these results show that the proposed model benefits from the integrated use of local feature refinement, global interaction modeling, and enhanced contextual fusion.

In addition to the component-level ablation analysis, a sensitivity analysis was conducted to further examine the influence of the learning rate. The tested values were 1 × 10^–4^, 1 × 10^–3^, and 1 × 10^–2^. Across these settings, the model achieved ACC values ranging from 95.16 ± 6.25% to 95.89 ± 5.55%, SEN values from 96.40 ± 2.86% to 96.70 ± 2.68%, SPE values from 94.39 ± 7.77% to 95.48 ± 6.36%, and AUC values from 98.74 ± 2.09% to 99.02 ± 1.72%. The learning rate used in the main experiments, 1 × 10^–3^, achieved the highest ACC, SPE, and AUC while maintaining high SEN, indicating a favorable overall balance among the evaluation metrics.

Finally, an event-level leave-one-seizure-event-out validation was conducted to further evaluate prediction-oriented alarm performance. As shown in Table 6, 11 patients satisfied the event-level inclusion criteria and were included in this analysis. The proposed DC-SA-EBiLSTM model achieved a mean event sensitivity of 95.96%, with an average FPR/h of 0.316 and a mean early warning time of 30.52 min.

**TABLE 6:** Event-level leave-one-seizure-event-out validation results.

Patient-ID	No. of test events	Event_sensitivity (%)	FPR/h	Early warning time (min)
Chb-01	6	100.0	0.000	29.16
Chb-03	5	100.0	0.036	31.26
Chb-05	5	100.0	0.906	28.04
Chb-06	9	88.89	0.177	32.15
Chb-09	4	100.0	0.413	23.21
Chb-10	6	83.33	0.256	32.52
Chb-13	6	83.33	0.666	32.52
Chb-16	7	100.0	0.571	32.52
Chb-20	5	100.0	0.050	32.52
Chb-21	4	100.0	0.397	32.52
Chb-23	5	100.0	0.000	29.30
**Mean**		**95.96**	**0.316**	**30.52**

Bold values indicate the mean summary results across all included patients.

At the patient level, 8 of the 11 patients achieved 100% event sensitivity, indicating that all held-out seizure events in these patients were successfully predicted within the predefined preictal interval. Chb-06, Chb-10, and Chb-13 showed lower event sensitivity, with values of 88.89, 83.33, and 83.33%, respectively. The false alarm rate also varied across patients. Chb-05 and Chb-13 showed relatively higher FPR/h values of 0.907 and 0.667, whereas Chb-01 and Chb-23 showed no false alarms in the event-level test setting. These results indicate that the proposed model achieved high event-level sensitivity overall, while false alarm control remained patient-dependent.

The mean early warning time was 30.52 min, suggesting that the model generally generated alarms before seizure onset within the predefined prediction interval. Together with the patient-specific ten-fold results, the event-level validation provides a stricter assessment of the model from an alarm-based seizure prediction perspective.

### 3.3 Discussion

To ensure a fair comparison, only studies that reported results on the same CHB-MIT dataset were included in Table 7. Wu et al. (2023) employed Compressed Sensing techniques to compress EEG signals, reducing the transmission bandwidth requirement, and used a CNN to reconstruct the compressed signals and predict seizures. Despite varying the compression ratio from 1/2 to 1/16, the prediction accuracy only dropped by 0.6%. In Rasheed et al.’s study (Rasheed et al., 2021a), the authors used a deep convolutional Generative Adversarial Network (GAN) to address the data imbalance problem and combined one-class SVM with CNN to successfully predict seizures. More recently, Jiang et al. (2023) proposed that changes in human EEG signal frequencies are related to seizures, and based on frequency-domain analysis, they used phase-amplitude coupling and machine learning to predict seizures. Notably, they adopted a patient-independent approach using 21 subjects from the CHB-MIT dataset, randomly selecting 17 for training and 4 for testing, ultimately achieving a maximum sensitivity rate of 91.7%.In Chen’s study (Chen et al., 2024) a spiking convolutional transformer framework was introduced, incorporating a SA to enhance feature recognition capabilities, ultimately achieving an accuracy of 93.1% on the CHB-MIT dataset. Qi et al. (2025) proposed an improved vision transformer model with EEG channel optimization for EEG-based seizure prediction. Compared with previous CHB-MIT-based studies, the proposed method provides a competitive feature-driven alternative for patient-specific seizure prediction. By combining compact handcrafted EEG descriptors with depthwise separable convolution, self-attention, and enhanced BiLSTM-based contextual fusion, the framework balances multiband feature representation and structured channel-wise modeling while avoiding direct raw-signal modeling. This design provides a practical basis for efficient seizure prediction model development.

**TABLE 7:** Comparison of the previous methods and proposed method on CHB-MIT dataset.

Papers	Year	Methods	Compare parameters (%)
Rasheed et al. (2021a)	2021	DCGAN, CESP	92.87 (SEN)
Wu et al. (2023)	2023	CS, CNN	γ 1/2, 90.3 (ACC)93.9 (SEN) γ 1/16, 89.7 (ACC)93.3 (SEN)
Jiang et al. (2023)	2023	Peak frequency, median frequency, RF	95.87 (ACC)95 (SEN) 95 (F1-SCORE)
Chen et al. (2024)	2024	Spiking conformer, SA	93.1 (ACC)96.8 (SEN) 89.5 (SPE)
Qi et al. (2025)	2025	vision transformer	93.65 (ACC)94.70 (SEN)92.78 (SPEC)
**This paper**	**2026**	**DWT, STD, RMS, HFD, FuEn, DC-SA-EBiLSTM**	**95.89 (ACC)96.70 (SEN)95.48 (SPE)**

Bold values indicate the results of the method proposed in this paper.

## 4 Conclusion

In this study, we proposed a feature-driven DC-SA-EBiLSTM framework for patient-specific seizure prediction. The method combines DWT-based multiband EEG descriptors with depthwise separable convolution, self-attention, and an enhanced BiLSTM-based fusion block to integrate complementary EEG feature information. Experiments on the CHB-MIT dataset showed that the proposed model achieved favorable performance across 21 patients, with an average ACC of 95.89%, SEN of 96.70%, SPE of 95.48%, and AUC of 99.02%. Baseline comparison, component-level ablation, and statistical analysis further supported the effectiveness of the complete framework.

In addition to conventional classification evaluation, event-level leave-one-seizure-event-out validation was conducted to assess alarm-based prediction performance. The model achieved an event sensitivity of 95.96%, an average false alarm rate of 0.316 FPR/h, and a mean early warning time of 30.52 min, indicating its potential for patient-specific seizure prediction. Future work will focus on external dataset validation, cross-patient generalization, improved false alarm control, and more explicit modeling of EEG spatial relationships.

## 5 Limitations and future work

Although the proposed DC-SA-EBiLSTM framework achieved favorable patient-specific seizure prediction performance on the CHB-MIT dataset, several limitations should be noted. First, the experiments were conducted on a single public dataset, and the evaluation was mainly patient-specific. Therefore, the generalizability of the proposed model to external datasets, different recording systems, and cross-patient prediction settings remains to be further investigated. Future work will evaluate the proposed framework on additional scalp EEG and intracranial EEG datasets and explore domain adaptation or transfer learning strategies for improving cross-patient robustness.

Second, although an event-level leave-one-seizure-event-out validation was added to provide a stricter alarm-based assessment, the analysis was still retrospective. Real-time seizure prediction may be affected by additional factors, including continuous data quality variation, online artifact contamination, and patient-specific changes over long-term monitoring. In addition, majority-class undersampling was used in the patient-specific experiments to reduce the dominance of interictal samples during training. Future studies may further investigate imbalance-aware learning strategies and evaluate whether they can improve false alarm control while maintaining sensitivity.

Third, the proposed model organizes EEG features according to a predefined CHB-MIT bipolar montage order to ensure consistent input representation. This design enables structured channel-wise modeling, but it does not explicitly encode the anatomical distances or graph topology among electrodes. Future work may incorporate topology-aware spatial modeling, such as graph-based EEG representations or distance-informed attention mechanisms, to better characterize neurophysiological relationships among channels.

## Data Availability

Publicly available datasets were analyzed in this study. This data can be found here: The dataset analyzed in this study is the publicly available CHB-MIT Scalp EEG Database hosted on PhysioNet: https://physionet.org/content/chbmit/1.0.0/; DOI: 10.13026/C2K01R.
